# Evolution of ART Medication Adherence Support and Monitoring Across IeDEA Sites: Results From Three Waves of Consortium‐Wide Site Assessment Surveys, 2017, 2020 and 2023

**DOI:** 10.1002/jia2.70197

**Published:** 2026-09-03

**Authors:** Ellen Brazier, Francesca Odhiambo, Jessie K. Edwards, Lauren Zalla, Charles Kasozi, Eugene Messou, Chantel Schreuder, Romanee Chaiwarith, María Fernanda Rodríguez, Irene Ayakaka, Marcel Zannou, Aimee Freeman, Peter Vanes Ebasone, Dina Muktiarti, Aggrey Semeere, Vohith Khol, Ephrem Mensah, Carina Cesar, Frank Tanser, Edwin Sang, Cuong Duy Do, Armel Poda, Annabelle Niyongabo, Yanink Caro Vega, Jonathan Arthur Colasanti, Idiovino Rafael, Patricia Lelo, Gad Murenzi, Denis Nash, the International Epidemiology Databases to Evaluate AIDS (IeDEA)

**Affiliations:** ^1^ Institute for Implementation Science in Population Health Graduate School of Public Health and Health Policy City University of New York New York New York USA; ^2^ KEMRI‐CMR‐RCTP Lumumba Sub‐County Hospital Grounds Kisumu Kenya; ^3^ Department of Epidemiology Gillings School of Global Public Health University of North Carolina Chapel Hill North Carolina USA; ^4^ Department of Epidemiology Johns Hopkins Bloomberg School of Public Health Baltimore Maryland USA; ^5^ Masaka Regional Referral Hospital Masaka Uganda; ^6^ CePReF Abidjan Côte d'Ivoire; ^7^ Desmond Tutu Health Foundation Cape Town South Africa; ^8^ Faculty of Medicine and Research Institute for Health Sciences Chiang Mai University Chiang Mai Thailand; ^9^ Fundación Arriarán Santiago Chile; ^10^ SolidarMed, Partnerships for Health Maseru Lesotho; ^11^ CNHU Cotonou Benin; ^12^ Bloomberg School of Public Health Johns Hopkins University Baltimore Maryland USA; ^13^ Clinical Research Education Networking and Consultancy (CRENC) Yaoundé Cameroon; ^14^ Faculty of Medicine Universitas Indonesia Cipto Mangunkusumo Hospital Jakarta Indonesia; ^15^ Infectious Diseases Institute College of Health Sciences Makerere University Kampala Uganda; ^16^ National Center for HIV/AIDS Dermatology & STDs Phnom Penh Cambodia; ^17^ Clinique Espoir Vie‐Togo Lome Togo; ^18^ Fundacion Huesped Buenos Aires Argentina; ^19^ Africa Health Research Institute Durban South Africa; ^20^ South African Centre for Epidemiological Modelling and Analysis (SACEMA) School of Data Science and Computational Thinking Stellenbosch University Stellenbosch South Africa; ^21^ Centre for Epidemic Response and Innovation School of Data Science and Computational Thinking Stellenbosch University Stellenbosch South Africa; ^22^ Academic Model Providing Access to Healthcare (AMPATH) Eldoret Kenya; ^23^ Bach Mai Hospital Hanoi Vietnam; ^24^ Department of Infectious Diseases Université Nazi Boni Bobo‐Dioulasso Burkina Faso; ^25^ Association Nationale de Soutien aux Séropositifs et malades du SIDA‐Santé PLUS (ANSS‐Santé PLUS) Bujumbura Burundi; ^26^ Departamento de Infectología Instituto Nacional de Ciencias Médicas y Nutrición, Salvador Zubirán Mexico City Mexico; ^27^ Division of Infectious Diseases Department of Medicine Emory University School of Medicine Atlanta Georgia USA; ^28^ SolidarMed Chiure Mozambique; ^29^ Department of Infectious Diseases Kalembelembe Pediatric Hospital Kinshasa Democratic Republic of the Congo; ^30^ College of Medicine and Health Sciences University of Rwanda Kigali Rwanda; ^31^ Goodlife Access Hospital Kigali Rwanda; ^32^ Department of Epidemiology and Biostatistics City University of New York Graduate School of Public Health and Health Policy New York New York USA

**Keywords:** adherence monitoring, antiretroviral therapy, differentiated service delivery, HIV, medication adherence support

## Abstract

**Introduction:**

Since 2013, global HIV treatment guidelines have included recommendations for medication adherence support and monitoring for people on antiretroviral therapy (ART). We examined the implementation of recommended strategies for adherence support and monitoring for adults with HIV through serial cross‐sectional structured surveys conducted by the International epidemiology Databases to Evaluate AIDS (IeDEA).

**Methods:**

We used data from surveys completed by HIV clinics across 43 countries in 2017 (*n* = 206), 2020 (*n* = 200) and 2023 (*n* = 214); 131 clinics completed all three surveys. We used descriptive statistics to examine clinic resources and routine adherence support and monitoring at each time point, stratified by country income level (i.e. low/middle‐income vs. high‐income countries [LMICs/HICs]), and trends among clinics completing all three surveys. We created composite measures of the level or intensity of routine provision of adherence aids/reminders, adherence support and adherence monitoring, ranging from “none” to “enhanced” levels. Among clinics participating in the 2023 survey, we also examined adherence aids and support routinely provided to clients eligible for intensified support versus all clients.

**Results:**

At each time point, surveyed clinics were predominantly in LMICs (68.4%−74.3%) and urban settings (65.0%−65.5%), situated within health centres (60.2%−64.0%) or regional/provincial or university hospitals (28.0%−33.5%). Among 131 clinics participating in all surveys, there was a decrease in the provision of multiple adherence readiness counselling sessions from 52.2% of clinics reporting ≥2 sessions before treatment initiation in 2017 to 26.0% in 2023, along with increased provision of 6‐month ART refills (from 11.5% to 40.5%). The provision of “enhanced” levels of adherence support to all clients decreased from 2017 to 2023 in both LMICs (64.6% to 28.1%) and HICs (34.3% to 8.6%); in 2023, substantial proportions of clinics reported that such support was routinely provided only to clients eligible for intensive adherence support. At each time point, higher proportions of clinics in LMICs reported “enhanced” adherence monitoring than in HICs (63.5%−70.8% vs. 31.4%−54.3%).

**Conclusions:**

While streamlined provision of ART adherence support may reflect the implementation of guidelines recommending the targeting of support towards clients with known or suspected adherence challenges, further research should examine how evolving clinic adherence support practices are associated with care retention and virologic suppression outcomes.

## 1 Introduction

Adherence to antiretroviral therapy (ART) is critical for reducing the risk of HIV disease progression among people living with HIV (PLHIV), as well as preventing the emergence of drug resistance, reducing onward transmission, and, ultimately, ending the HIV epidemic [1−3].

Since 2013, the World Health Organization (WHO) has recommended strategies to support and optimize ART adherence and retention, including education, counselling, peer support, adherence aids and reminders, as well as interventions to address mental health and substance use, financial barriers and issues related to food insecurity that affect adherence [4]. Additionally, WHO has recommended various adherence assessment measures, ranging from simple, low‐cost self‐report measures, pharmacy refill checks and pill counts, which may overestimate ART adherence, to more accurate—and costly—measures, such as electronic medication event monitoring systems (MEMS) devices and routine viral load (VL) monitoring [5−11]. WHO's guidance on adherence support and monitoring has remained largely unchanged in subsequent guidelines for universal treatment of all PLHIV [12], differentiated service delivery (DSD) for patients stable on ART [13], same‐day and rapid ART initiation among those diagnosed with HIV [14, 15], and the rollout of new integrase inhibitor‐based regimens, which are more tolerant of sub‐optimal adherence [16].

Despite WHO's longstanding recommendations, apart from single‐country studies evaluating intervention effectiveness [17], there is little data on the implementation of recommended strategies for adherence support and monitoring in HIV service delivery around the globe—information that is important from an implementation research standpoint and for examining how adherence support and monitoring are associated with HIV care outcomes, such as retention and viral suppression. Accordingly, we sought to describe clinic resources and practices related to adherence support and monitoring among a diverse global cohort of HIV clinics participating in the International epidemiology Databases to Evaluate AIDS (IeDEA) and to examine trends in adherence support and monitoring in both high‐ and low‐income settings.

## 2 Methods

### 2.1 Data Sources

Data were from serial cross‐sectional site surveys conducted by IeDEA in 2017, 2020 and 2023. IeDEA is a global consortium comprising HIV care and treatment sites across seven geographic regions: the Asia‐Pacific; Latin America and the Caribbean (CCASAnet); Central Africa; East Africa; Southern Africa; West Africa; and North America (NA‐ACCORD) [18]. As discussed elsewhere [19], IeDEA regularly conducts surveys of participating clinics to document HIV service availability across sites and over time. IeDEA's consortium‐wide site surveys are structured questionnaires, self‐administered in English or French by clinic staff involved in HIV care, using an electronic REDCap survey link [20, 21] or a printed questionnaire subsequently entered into REDCap by IeDEA research coordinators. The survey is distributed to clinic staff familiar with HIV‐related services who are assured that there are no incorrect answers to the survey's questions about routine practices at their site. Designated as non‐human subjects research per 45 CFR §46.102(l) by the Vanderbilt University Medical Center (VUMC) Institutional Review Board (IRB#161017, IRB#200013, IRB#230843), IeDEA's survey response rates have ranged from 91% to 96% [19, 22, 23].

Covering all IeDEA regions, our study focused on survey responses from clinics providing care to adults living with HIV across 43 countries in 2017 (*N* = 206), 2020 (*N* = 200) and 2023 (*N* = 214). Each survey included questions about clinic resources for adherence support and monitoring, including staffing, laboratory testing capacity and adherence aids/reminders (e.g. pillboxes, blister packs, visual aids/reminders or electronic devices/reminders), as well as routine practices related to ART adherence support and monitoring (Table 1). The 2023 survey included an additional question about eligibility criteria for intensified adherence support and explored whether adherence aids and adherence support interventions were routinely provided to “all patients” or “only to patients eligible for intensified or enhanced adherence support.”

**TABLE 1:** Survey domains related to ART adherence support and monitoring.

Survey domain	Items
Staff available daily	Adherence counsellors Peer educators/counsellors Outreach staff
Laboratory testing capacity	Viral load testing services availability, onsite or off‐site Genetic testing for drug resistance, onsite or off‐site Turnaround times for viral load testing results
Adherence aids/reminders	Client education materials Pill boxes or blister packs Visual reminders/aids (e.g. calendars, diaries, checklists, etc.) Electronic reminder devices (e.g. beepers, phone apps, alarm clocks or wrist watches)
Routine clinic practices related to ART adherence support	Number of ART readiness counselling sessions ART refill frequency for established/stable clients Individual counselling^a^ Group counselling^a^ Peer counselling^a^ Support groups^a^ ART adherence clubs^a^ Cognitive behavioural/mental health intervention^a^ Other adherence support (e.g. cash incentives, food supplements or directly observed treatment)^a^
Routine practices related to adherence monitoring	Client self‐report Review of pharmacy pickups Pill counts Electronic monitoring devices (e.g. MEMS devices) Routine viral load monitoring
Clients eligible for intensified adherence support^a^	Self‐reported adherence problems Suspected adherence problems/irregular visits Detectable viral loads Viral load >1000 copies/mL New opportunistic infections New ART initiators with advanced HIV disease

^a^ 2023 survey explored whether adherence support interventions were provided to all clients or only to clients eligible for intensified adherence support.

### 2.2 Statistical Analysis

We used descriptive statistics to characterize clinics participating in each survey, along with resources for adherence support and monitoring at each time point. We categorized country income levels as low/middle‐income countries (LMICs) versus high‐income countries (HICs) using World Bank classifications [24]. Among clinics participating in all three surveys, we examined trends over time, using Cochran−Armitage trend and Cochran−Mantel−Haenszel tests. In supplementary analyses, we examined differences between LMICs and HICs, using chi‐squared tests, and we examined trends over time in each income setting.

Using data from clinics participating in all three surveys, we created three composite measures to summarize the level or intensity of adherence aids/reminders, adherence support and adherence monitoring routinely provided to all clients on ART at each survey time point—and, additionally, in 2023 to clients eligible for intensified support—stratified by country income level. The provision of adherence aids was categorized as: None; Minimal (educational materials only); Moderate (any reminder aid/device); or Enhanced (>1 reminder aid or device). Routine adherence support was categorized as: None; Minimal (individual or group/peer counselling only); Moderate (individual or group/peer counselling plus other incentive/support—e.g. cash incentives, food supplements or directly observed treatment); or Enhanced (individual counselling plus group/peer counselling plus other incentive/support). Routine adherence monitoring was categorized as: None; Minimal (self‐reported adherence or pill counts or pharmacy refill checks); Moderate (VL monitoring alone or VL monitoring plus either self‐report or pill counts or pharmacy refill checks); or Enhanced (VL monitoring or electronic device monitoring plus ≥2 other adherence assessment measures—e.g. self‐report, pill counts or pharmacy refill checks).

All statistical analyses were performed using SAS 9.4 (SAS Institute, Cary, NC), with an alpha of 0.05 used in tests of statistical significance.

## 3 Results

Reflecting the composition of the IeDEA consortium, the majority of clinics were in LMICs (68.4%−74.3%) and in urban settings (65.0%−65.5%), primarily in IeDEA's East Africa, Asia‐Pacific and North America regions (Table 2). Most clinics (60.2%−64.0%) were located within mid‐level health facilities (i.e. health centres) or regional, provincial or university hospitals (28.0%−33.5%), with few located within district hospitals (6.3%−8.0%). The distribution and characteristics of 131 clinics that participated in all three surveys were similar to those of clinics participating in each survey.

**TABLE 2:** Characteristics of sites completing IeDEA's consortium‐wide surveys, 2017–2023.

	2017	2020	2023	Sites participating in all surveys
Site characteristic—*N* (%)	*N* = 206	*N* = 200	*N* = 214	*N* = 131
IeDEA region				
Asia‐Pacific	38 (18.4)	37 (18.5)	37 (17.3)	27 (20.6)
Central Africa	19 (9.2)	21 (10.5)	24 (11.2)	19 (14.5)
East Africa	56 (27.2)	74 (37.0)	75 (35.0)	46 (35.1)
Southern Africa	35 (17.0)	25 (12.5)	30 (14.0)	8 (6.1)
West Africa	6 (2.9)	6 (3.0)	7 (3.3)	3 (2.3)
Latin America and Caribbean	11 (5.3)	7 (3.5)	7 (3.3)	7 (5.3)
North America	41 (19.9)	30 (15.0)	34 (15.9)	21 (16.0)
Country income level				
Lower‐middle income^a^	141 (68.4)	148 (74.0)	159 (74.3)	96 (73.3)
High income^b^	65 (31.6)	52 (26.0)	55 (25.7)	35 (26.7)
Clinic location				
Rural/mostly rural	72 (35.0)	69 (34.5)	74 (34.6)	39 (29.8)
Urban/mostly urban	134 (65.0)	131 (65.5)	140 (65.4)	92 (70.2)
Facility level				
Health centre	124 (60.2)	126 (63.0)	137 (64.0)	74 (56.5)
District hospital	13 (6.3)	16 (8.0)	17 (7.9)	8 (6.1)
Regional, provincial or university hospital	69 (33.5)	58 (29.0)	60 (28.0)	49 (37.4)

^a^ Argentina, Burundi, Benin, Burkina Faso, Brazil, Cambodia, Cameroon, China, Cote d'Ivoire, Congo, Democratic Republic of Congo, Haiti, Honduras, India, Indonesia, Kenya, Lesotho, Malawi, Mexico, Mozambique, Nigeria, Peru, Philippines, Rwanda, Senegal, South Africa, Tanzania, Thailand, Togo, Uganda, Vietnam, Zambia and Zimbabwe.

^b^ Australia, Canada, Chile, Japan, Korea, New Zealand, Singapore, Taiwan and the United States.

### 3.1 Clinic Resources Related to ART Adherence Support and Monitoring

Clinic resources related to routine adherence support and monitoring at each time point are shown in Table 3 and disaggregated by country income level in Table S1. At each time point, most sites reported having an adherence counsellor available daily (69.4%−76.4%). Fewer clinics reported daily availability of peer educators/counsellors (47.1%−59.3%) or outreach staff (47.1%−49.5%). The availability of these cadres was similar among the subset of clinics participating in all three surveys, with little change over time. At each time point, the availability of adherence counsellors and peer counsellors was higher at clinics in LMICs than HICs (Table S1).

**TABLE 3:** Clinic resources for adherence support and monitoring by survey year, and trends among 131 clinics participating in all three surveys.

	All clinics			Clinics participating in all three surveys (*N* = 131)			
Clinic resources	2017 *N* = 206	2020 *N* = 200	2023 *N* = 214	2017	2020	2023	Time trends (2017−2023)^a^
Staffing (available every day clinic is open)	*n* (%)	*n* (%)	*n* (%)	*n* (%)	*n* (%)	*n* (%)	
Adherence counsellor	143 (69.4)	153 (76.5)	163 (76.2)	100 (76.3)	104 (79.4)	104 (79.4)	0.548
Peer educators/counsellors	97 (47.1)	120 (60.0)	127 (59.3)	76 (58.0)	84 (64.1)	76 (58.0)	1.00
Outreach staff	97 (47.1)	97 (48.5)	106 (49.5)	62 (47.3)	66 (50.4)	60 (45.8)	0.805
Number of pre‐ART counselling sessions conducted							
0	20 (9.7)	20 (10.0)	26 (12.1)	12 (9.2)	12 (9.2)	13 (9.9)	<0.001^b^
1	93 (45.1)	84 (42.0)	132 (61.7)	48 (36.6)	59 (45.0)	84 (64.1)	
2 or more sessions	93 (45.1)	50 (25.0)	55 (25.7)	71 (54.2)	36 (27.5)	34 (26.0)	
Unknown/not reported	0 (0)	46 (23.0)	1 (0.5)	0 (0)	24 (18.3)	0 (0)	
ART refill frequency for stable clients							
Monthly	27 (13.1)	26 (13.0)	9 (4.2)	19 (14.5)	15 (11.5)	6 (4.6)	<0.001^b^
Every 3 months	128 (62.1)	126 (63.0)	89 (41.6)	78 (59.5)	81 (61.8)	60 (45.8)	
Every 6 months	23 (11.2)	28 (14.0)	97 (45.3)	15 (11.5)	19 (14.5)	53 (40.5)	
Other/unknown	28 (13.6)	20 (10.0)	19 (8.9)	19 (14.5)	16 (12.2)	12 (9.2)	
Viral load testing services							
No onsite viral load testing	126 (61.2)	99 (49.5)	91 (42.5)	73 (55.7)	59 (45.0)	47 (35.9)	0.001
Onsite viral testing (within facility or clinic)	80 (38.8)	101 (50.5)	123 (57.5)	58 (44.3)	72 (55.0)	84 (64.1)	
Turnaround time for viral load test results							
0−7 days	80 (38.8)	89 (44.5)	102 (47.7)	54 (41.2)	61 (46.6)	62 (47.3)	0.003^b^
8−14 days	81 (39.3)	40 (20.0)	55 (25.7)	55 (42.0)	25 (19.1)	41 (31.3)	
15−30 days	36 (17.5)	54 (27.0)	48 (22.4)	18 (13.7)	36 (27.5)	24 (18.3)	
>30 days	7 (3.4)	15 (7.5)	7 (3.3)	3 (2.3)	8 (6.1)	2 (1.5)	
Not available	2 (1.0)	2 (1.0)	2 (0.9)	1 (0.8)	1 (0.8)	2 (1.5)	
Genetic testing for drug resistance							
No onsite drug resistance testing	111 (53.9)	141 (70.5)	131 (61.2)	73 (55.7)	93 (71.0)	75 (57.3)	0.800
Onsite drug resistance (within facility or clinic)	95 (46.1)	59 (29.5)	83 (38.8)	58 (44.3)	38 (29.0)	56 (42.7)	

Abbreviation: ART, antiretroviral therapy.

^a^ Cochran−Armitage trend test, unless otherwise noted.

^b^ Cochran−Mantel−Haenszel test.

Overall, clinics reported a progressive streamlining of ART readiness counselling from 2017 to 2023 (Table 3), with fewer sites reporting that two or more counselling sessions were conducted prior to treatment initiation (from 45.1% in 2017 to 25.7% in 2023 among all sites; from 54.2% to 26.0% among sites participating in all three surveys) and corresponding increases in reporting of one ART readiness counselling session only. At each time point, higher proportions of clinics in HICs than LMICs reported that no ART readiness counselling sessions were routinely provided prior to treatment initiation (27.7%−38.2% in HICs vs. 1.4%−3.1% in LMICs) (Table S1).

ART refill frequencies for stable clients also changed over time; 11.2%−14.0% of clinics reported 6‐month refills in 2017 and 2020, compared with 45.3% in 2023, and the proportion reporting 3‐month refills decreased from 62.1%−63.0% in 2017 and 2020 to 41.6% in 2023. Similar trends were observed among clinics participating in all three surveys. These temporal changes primarily occurred in LMICs, where 6‐month refills for stable clients on ART increased from 5.7% in 2017 to 48.4% in 2023.

There was a modest increase reported in onsite availability of VL testing, from 38.8% in 2017 to 57.5% in 2023 (44.3%−64.1% at clinics participating in all three surveys). The increase in onsite availability of VL testing was particularly pronounced at HIC clinics (from 67.7% to 100% in 2023), compared with LMIC clinics (from 25.5% to 42.8%). Less than half of LMIC clinics (20.6%−34.0%) reported VL test turnaround times of 0–7 days, compared with 78.5%−87.3% of HIC clinics. Onsite availability of genetic testing for HIV drug resistance also differed by country income level (HICs: 78.8%−95.4% vs. LMICs: 12.2%−23.4%), with little change over time in either setting.

### 3.2 Adherence Aids, Support Interventions and Monitoring Practices

Table 4 shows trends in the provision of adherence aids, support interventions and adherence monitoring. Trends by country income level are shown in Table S2. At each time point, less than half of clinics reported routinely providing client education materials (45.6%−43.9%); visual aids/reminders (32.5%−33.6%); or electronic reminder devices (21.4%−21.0%) to support ART adherence. Trends in the provision of such adherence aids/reminders differed by country income setting (Table S2); in LMICs, routine provision of client education materials on adherence increased modestly (from 34.8% in 2017 to 47.8% in 2023), whereas the proportion of HIC clinics providing such materials to all clients decreased (from 69.2% to 32.7%). Routine provision of pill boxes/blister packs also decreased at HIC clinics (from 76.9% to 20.0%), with no change observed among LMIC clinics (from 24.8% to 25.2%).

**TABLE 4:** Adherence aids, support and monitoring practices by survey year, and trends among 131 clinics participating in all three surveys.

Adherence aids, interventions and monitoring practices	All clinics			Clinics participating in all three surveys (*N* = 131)			
	2017	2020	2023	2017	2020	2023	Time trends (2017−2023)^b^
	*N* = 206	*N* = 200	*N* = 214				
ART adherence aids routinely provided to all clients	*n* (%)	*n* (%)	*n* (%)	*n* (%)	*n* (%)	*n* (%)	
None	49 (23.8)	39 (19.5)	78 (36.4)	18 (13.7)	26 (19.8)	50 (38.2)	<0.001
Client education materials	94 (45.6)	98 (49.0)	94 (43.9)	68 (51.9)	68 (51.9)	57 (43.5)	0.174
Pill box/blister packs	85 (41.3)	104 (52.0)	51 (23.8)	57 (43.5)	72 (55.0)	29 (22.1)	<0.001
Visual aids/reminders	67 (32.5)	70 (35.0)	72 (33.6)	48 (36.6)	42 (32.1)	40 (30.5)	0.294
E‐devices/ reminders	44 (21.4)	72 (36.0)	45 (21.0)	39 (29.8)	53 (40.5)	28 (21.4)	0.140
Routine adherence support interventions for all clients							
None	10 (4.9)	3 (1.5)	44 (20.6)	7 (5.3)	1 (0.8)	26 (19.8)	<0.001
Individual counselling	184 (89.3)	190 (95.0)	130 (60.7)	120 (91.6)	125 (95.4)	81 (61.8)	<0.001
Group counselling	108 (52.4)	104 (52.0)	75 (35.0)	71 (54.2)	69 (52.7)	39 (29.8)	<0.001
Peer counselling/support group or ART club	108 (52.4)	110 (55.0)	88 (41.1)	76 (58.0)	69 (52.7)	52 (39.7)	<0.001
Cognitive behavioural/mental health intervention	69 (33.5)	96 (48.0)	39 (18.2)	47 (35.9)	56 (42.7)	19 (14.5)	<0.001
Other support^a^	74 (35.9)	34 (17.0)	21 (9.8)	49 (37.4)	18 (13.7)	12 (9.2)	<0.001
Routine adherence monitoring practices							
None	1 (0.5)	0 (0)	0 (0)	0 (0)	0 (0)	0 (0)	
Client self‐report	160 (77.7)	164 (82.0)	175 (81.8)	116 (88.5)	111 (84.7)	107 (81.7)	0.120
Review pharmacy pickups	108 (52.4)	132 (66.0)	155 (72.4)	77 (58.8)	92 (70.2)	88 (67.2)	0.153
Pill counts	96 (46.6)	104 (52.0)	121 (56.5)	62 (47.3)	66 (50.4)	67 (51.1)	0.537
Electronic monitoring (MEMS caps)	3 (1.5)	38 (19.0)	12 (5.6)	2 (1.5)	20 (15.3)	5 (3.8)	0.348
Routine viral load monitoring	171 (83.0)	169 (84.5)	181 (84.6)	113 (86.3)	115 (87.8)	113 (86.3)	1.000
Self‐reported adherence measures							
None	46 (22.3)	37 (18.5)	20 (9.3)	15 (11.5)	21 (16.0)	13 (9.9)	<0.001^c^
Unstructured measures only	56 (27.2)	52 (26.0)	36 (16.8)	32 (24.4)	33 (25.2)	19 (14.5)	
Structured measures only	77 (37.4)	86 (43.0)	62 (29.0)	62 (47.3)	58 (44.3)	36 (27.5)	
Structured and unstructured measures	27 (13.1)	25 (12.5)	96 (44.9)	22 (16.8)	19 (14.5)	63 (48.1)	
Clients eligible for intensified adherence support							
None	—	—	1 (0.5)	—	—	0 (0)	
Self‐reported adherence problems	—	—	181 (84.6)	—	—	110 (84.0)	
Suspected adherence problems/irregular visits	—	—	193 (90.2)	—	—	120 (91.6)	
Detectable viral loads	—	—	145 (67.8)	—	—	92 (70.2)	
Viral load >1000 copies/mL	—	—	61 (28.5)	—	—	35 (26.7)	
New opportunistic infections	—	—	142 (66.4)	—	—	86 (65.6)	
New ART initiators with advanced HIV disease	—	—	141 (65.9)	—	—	83 (63.4)	

Abbreviations: ART, antiretroviral therapy; MEMS, medication event monitoring system; n/a, not applicable.

^a^ Other support: Cash incentives, food supplements or directly observed treatment.

^b^ Cochran−Armitage trend test unless otherwise noted.

^c^ Cochran−Mantel−Haenszel test.

Among all clinics, the proportion reporting that no adherence support interventions were routinely provided to all clients on ART ranged from 4.9% in 2017 to 20.6% in 2023, with similar trends observed among clinics participating in all three surveys (from 5.3% to 19.8%), LMIC clinics (from 2.1% to 14.5%) and HIC clinics (from 10.8% to 38.2%). Among all clinics, the proportion reporting routine provision of individual adherence counselling to all clients also decreased from 89.3% in 2017 to 60.7% in 2023, with similar decreases among sites participating in all three surveys and in both LMIC and HIC settings. Decreases were also reported in routine provision to all clients of: group adherence counselling (from 52.4% in 2017 to 35.0% in 2023); peer counselling, support groups and ART clubs for adherence support (from 52.4% to 41.1%); cognitive behavioural therapy/mental health interventions for adherence (from 33.5% to 18.2%) and other adherence support interventions, such as cash incentives, food supplements and directly observed treatment (from 35.9% to 9.8%)—most of which were more prevalent among LMIC clinics than HIC clinics at each time point (Table S2).

Most sites reported using routine VL testing for adherence monitoring (83.0%−84.6%), with little difference by country income level or change over time (Table 4). Most (77.7%−81.8%) also relied on self‐reported adherence, and increasing proportions of clinics reported using both structured and unstructured self‐reported adherence assessment measures. About half reported using pill counts (49.6%−56.4%) for adherence monitoring, and 52.4%−72.4% reported review of pharmacy pick‐ups, which increased in LMICs and decreased in HICs from 2017 to 2023 (Table S2). Use of electronic MEMS devices was low at all time points (1.5%−5.6%).

### 3.3 Trends in Routine Adherence Support and Monitoring

**FIGURE 1:**
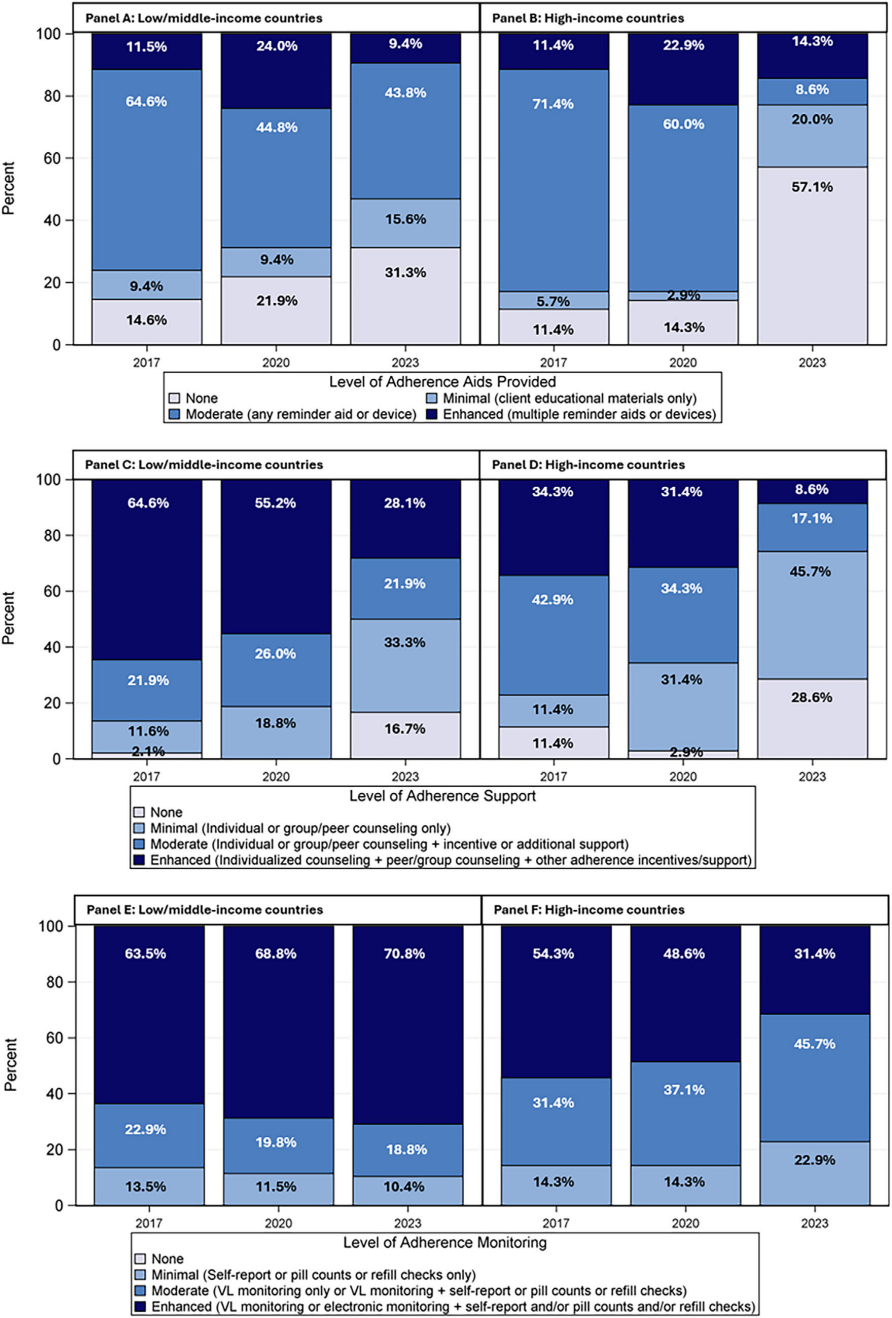
Trends in adherence support and monitoring among 131 clinics participating in all surveys.

Trends for composite measures of adherence support and monitoring among clinics participating in all three surveys are shown in Figure 1, stratified by country income level. In LMICs (Panel A) and HICs (Panel B), less than one‐quarter reported providing “enhanced” adherence aids (i.e. multiple types of aids, such as pillboxes, blister packs, visual aids/reminders or electronic devices/reminders) at any time point, and there was a substantial increase in the proportion of clinics reporting that no adherence aids were routinely provided to all clients (LMICs: 14.6% in 2017 to 31.3% in 2023, *p*<0.002; HICs: 11.4%−57.1%, *p*<0.001).

Trends related to the level of adherence support interventions are shown in Panels C and D. Decreasing proportions of clinics reported routinely providing “enhanced” levels of adherence support (i.e. individual counselling + group/peer counselling + other incentive/support), from 64.6% to 28.1% (*p*<0.001) among LMIC clinics and from 34.3% to 8.6% (*p*<0.001) among HIC clinics. At each time point, routine provision of “enhanced” levels of adherence support to all patients was almost two times higher among LMIC clinics than in HICs.

The majority of LMIC clinics (63.5%−70.8%) reported routine provision of “enhanced” levels of adherence monitoring (VL monitoring or electronic medical monitoring plus ≥2 other adherence assessment measures) at each time point, compared with 31.4%−54.3% of HIC clinics (Panels E and F). Reporting of “minimum” levels of adherence monitoring (self‐reported adherence, pill counts or pharmacy refill checks without VL monitoring) was low at all time points in LMICs (13.4% in 2017 to 10.4% in 2023), with a small, non‐significant increase reported by HIC clinics (14.3%−22.9%).

### 3.4 Adherence Aids and Support for Eligible Clients in 2023

In response to new questions explored in the 2023 survey, almost all sites reported that certain clients were eligible for intensified adherence support (Table 4), including clients with: self‐reported adherence problems (84.6%), suspected adherence problems or missed visits (90.2%), detectable VLs (67.8%), VLs >1000 copies/mL (28.5%), new opportunistic infections (66.4%); and advanced HIV disease at ART initiation (65.9%). There were no substantive differences in eligibility criteria reported by LMIC and HIC clinics (Table S2) apart from new onset of opportunistic infections (LMICs: 72.3% vs. HICs: 49.1%).

**FIGURE 2:**
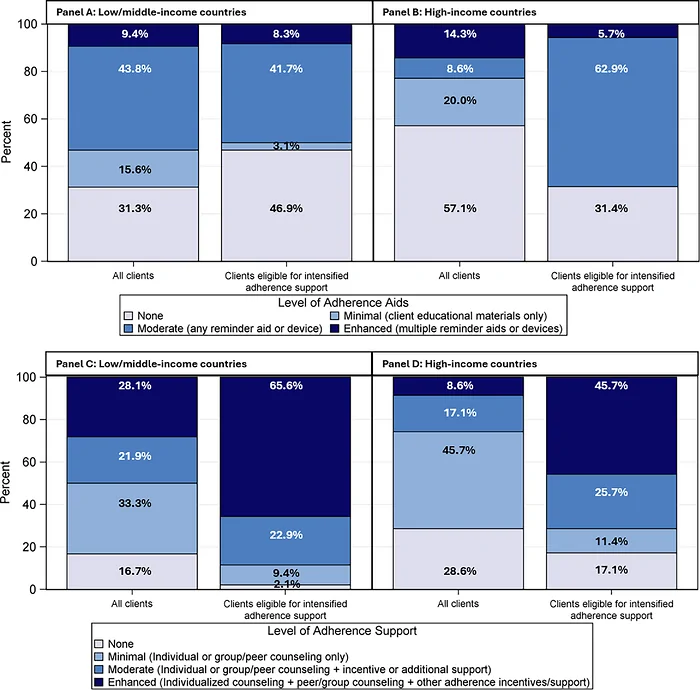
Level of adherence aids and support provided to all clients versus eligible clients in 2023 at 131 clinics participating in all surveys.

There were substantial differences in adherence aids and support provided to clients eligible for intensified adherence support in 2023 versus all clients on ART (Figure 2). In LMICs (Panel A), the provision of “moderate” or “enhanced” levels of adherence aids to eligible clients was comparable to that provided to all clients on ART. In contrast, HIC clinics (Panel B) were more likely to report providing “moderate” or “enhanced” adherence aids/reminders to clients eligible for intensified support than to all clients.

Two‐thirds of LMIC clinics reported providing “enhanced” levels of adherence support only to ART clients eligible for intensified support in 2023 (Panel C), whereas 28.1% reported providing such support to all clients. Among HIC clinics (Panel D), 45.7% and 25.7%, respectively, reported providing “enhanced” or “moderate” levels of adherence support to eligible clients only, whereas 8.6% and 17.1%, respectively, reported providing such support to all clients.

## 4 Discussion

With data from HIV clinics in 43 countries that participated in IeDEA's serial cross‐sectional consortium‐wide surveys in 2017, 2020 and 2023, we examined the implementation of recommended strategies for adherence support and monitoring in HIV service delivery in LMICs and HICs. At each survey time point, clinics in LMICs were better staffed for adherence support, compared with clinics in HICs, and they reported offering a broader range of adherence support interventions—from individualized counselling, group counselling and peer support groups to adherence aids and reminder devices—than those in HICs. Composite measures of adherence support and monitoring also indicated that the provision of “moderate” and “enhanced” levels of adherence support and monitoring were generally higher among clinics in LMICs than HICs.

We observed decreases in ART adherence resources, support and monitoring over time, in both LMIC and HIC settings, including decreases in the number of ART readiness counselling sessions conducted, as well as the provision of adherence reminder aids/devices and interventions, such as adherence counselling and support groups. Observed decreases in routine provision of intensive levels of adherence support to all clients may potentially reflect the adoption and scale‐up of WHO's 2016 and 2021 recommendations on DSD—namely the provision of client‐centred care, tailored to the preferences and needs of individuals served, as opposed to “one‐size fits all” models of HIV care [13, 14, 25]. This may be particularly true in LMICs, which generally rely on WHO recommendations in developing their own clinical guidelines and policies [26, 27]. Consistent with calls for accelerated adoption of DSD and multi‐month dispensing in response to the COVID‐19 pandemic [28] and other research [29, 30], we observed substantial increases in reported provision of 6‐month ART refills to stable clients on ART in LMICs between 2017 and 2023. Additionally, while we observed decreases in reported provision of “enhanced” adherence support to all clients over time, responses to our 2023 survey suggested that in both LMICs and HICs, these more intensive and multifaceted adherence support interventions are being targeted to clients with greatest needs—that is those with known or suspected adherence challenges, advanced HIV disease, or signs of viraemia or treatment failure. Observed decreases in the routine provision of multifaceted adherence support to all clients may also reflect programmatic responses to decreasing financing for the HIV response since its peak in 2014 [31] along with competing demands and financial shocks related to the COVID‐19 pandemic [32]. Additionally, temporal changes in clinic resources and practices related to routine adherence support may be related to the rollout of new, more potent ART regimens, such as second‐generation integrase strand transfer inhibitor (INSTI)‐containing regimens (e.g. dolutegravir or bictegravir); HIV clinics may be streamlining their routine adherence support because these regimens are known to have a high genetic barrier to HIV drug resistance, limited drug−drug interactions and toxicity, and greater tolerability and ease of use [33−36].

It is notable that at each time point, more than 10% of clinics did not report VL monitoring among routine adherence assessment practices, including 27% of HIC clinics in 2023, where on‐site VL testing capacity was universal and where the HIV epidemic is often concentrated among hard‐to‐serve populations [32, 37, 38] for whom objective measures of adherence may be particularly critical. In LMICs, reliance on measures other than VL monitoring may reflect gaps in VL testing capacity—gaps that persist in the era of universal HIV treatment [39, 40]. With less than half of clinics in LMICs reporting on‐site VL testing in 2023 and more than one‐third reporting turnaround times of 2 weeks or more for VL test results, gaps in VL and drug resistance testing capacity may impede timely identification of clients with adherence challenges and those with treatment failure.

Our study has several limitations. Importantly, as IeDEA surveys do not collect client‐level data, we cannot ascertain clients’ receipt of adherence support, and we cannot examine how clinic practices are associated with HIV care outcomes, such as retention on ART and viral suppression. Second, our study relied on adherence‐related questions included in structured surveys addressing multiple aspects of HIV‐related care. Survey questions did not solicit detailed information about the duration or number of adherence counselling sessions provided to clients, nor did they explore the training of personnel involved in adherence support. Accordingly, there may be substantial variation in the level of adherence support provided by clinics reporting the provision of individual or group counselling only. Further, it is possible that clinics reporting only individual counselling deliver more effective adherence support than clinics reporting multiple adherence support interventions.

Third, clinics participating in IeDEA's surveys change over time. Data for our study came from 286 unique clinics, including 131 clinics that participated in all three surveys. While compositional changes at each survey time point could potentially explain observed changes in clinic resources and practices, the subset of 131 clinics participating in all three surveys were similar to our overall sample, and trends among this subset were consistent with those among the full sample. Additionally, although our study relied primarily on questions explored in all three surveys, questions about clients eligible for intensified adherence support and the types of adherence aids and support provided to all clients versus eligible clients were introduced in 2023. Accordingly, we were unable to examine trends in the provision of differentiated adherence support over time, and we could not establish when participating clinics may have begun targeting adherence support to clients with known or suspected adherence challenges.

Finally, as described elsewhere [19], IeDEA's surveys are completed via self‐report. While responses may be subject to social desirability and recall biases, those completing IeDEA's surveys are encouraged to describe their day‐to‐day practices, and it is unlikely that response biases have substantially changed over time. Clinics participating in IeDEA may not be representative at country or regional levels as they are predominantly located in urban areas; with substantial numbers of regional, provincial and university hospitals providing specialized care and/or serving sizable client populations, IeDEA data likely reflect practices at better‐resourced health facilities, particularly in LMIC settings, suggesting that ongoing gaps in VL and resistance testing capacity in LMIC settings may be underestimated in our surveys.

These limitations notwithstanding, IeDEA's consortium‐wide surveys have high participation rates (>90%) [19, 41] and provide a window into HIV treatment resources and practices across diverse settings and the implementation of evidence‐based practices and WHO guidelines. Our composite measures of adherence support and monitoring suggest important capacity differences between LMICs and HICs, along with shifts in practices over time—shifts that merit further investigation through analyses of patient‐level data to understand how adherence support and monitoring practices are associated with care retention and viral suppression outcomes.

## 5 Conclusions

Data from serial cross‐sectional surveys completed by HIV clinics participating in IeDEA provide insights into the implementation of recommended adherence support and monitoring strategies. Study findings illuminate shifts in ART adherence support and monitoring in the era of universal treatment, suggesting increased targeting of adherence support towards clients with known or suspected adherence challenges in both LMICs and HICs, alongside persistent gaps in essential laboratory testing capacity in LMICs. Findings also highlight priorities for further research, including qualitative research on the factors contributing to observed trends in adherence support and exploration of the associations between clinic‐level practices and client‐level outcomes, including care retention and viral suppression, both before and after the introduction of DSD and the rollout of dolutegravir and other INSTI‐based regimens.

## Author Contributions

The International epidemiology Databases to Evaluate AIDS (IeDEA) coordinated the site‐level surveys. DN obtained funding. FO, RC, CK, EM, CS, DM, MFR, IA, MZ, AF, PVE, AS, VK, EM, CC, FT, ES, CDD, AP, AN, YCV, IR, GM and PL contributed to acquisition, analysis or interpretation of data. EB and DN conceived and designed this analysis. EB performed the statistical analysis, interpreted findings and drafted the manuscript. DN, LZ, JKE, FO, RC, CK, EM, CS, DM, MFR, IA, MZ, AF, PVE, AS, VK, EM, CC, FT, ES, CDD, AP, AN, YCV, IR, GM and PL critically reviewed the manuscript for important intellectual content. All authors read and approved the final manuscript.

## Funding

The International Epidemiology Databases to Evaluate AIDS (IeDEA) is supported by the U.S. National Institutes of Health’s (NIH) National Institute of Allergy and Infectious Diseases, the *Eunice Kennedy Shriver* National Institute of Child Health and Human Development, the National Cancer Institute, the National Institute of Mental Health, the National Institute on Drug Abuse, the National Heart, Lung, and Blood Institute, the National Institute on Alcohol Abuse and Alcoholism, the National Institute of Diabetes and Digestive and Kidney Diseases, and the Fogarty International Center: *Asia‐Pacific*, U01AI069907; *CCASAnet*, U01AI069923; *Central Africa*, U01AI096299; *East Africa*, U01AI069911; *NA‐ACCORD*, U01AI069918; *Southern Africa*, U01AI069924; *West Africa*, U01AI069919. Informatics resources are supported by the Harmonist project, R24AI124872.

## Conflicts of Interest

Authors (EB, DN, JKE, EM, AF, AS, CC and YCV) report NIH grants to their institutions, and DN reports institutional grants from NYC Department of Health, New York State AIDS Institute and Pfizer, Inc. All authors have declared no conflicts of interest.

## Disclaimer

This work is solely the responsibility of the authors and does not necessarily represent the official views of any of the institutions mentioned above. This manuscript is the result of funding in whole or in part by the NIH. It is subject to the NIH Public Access Policy. Through acceptance of this federal funding, NIH has been given a right to make this manuscript publicly available in PubMed Central upon the Official Date of Publication, as defined by NIH.

## Supporting information




**Table S1**: Clinic resources for adherence support and monitoring by survey time point and country income level.
**Table S2**: Adherence aids, support and monitoring practices by survey time point and country income level

**Text S1**: Survey acknowledgements.

## Data Availability

Those interested in accessing IeDEA data for research purposes may contact the corresponding author or review IeDEA guidance available at: https://www.iedea.org/resources/multiregional‐research‐sops‐templates/ and contact the IeDEA Executive Committee (https://www.iedea.org/working‐groups/executive‐committee/).
