# Supplementary material for: Evolution of ART Medication Adherence Support and Monitoring Across IeDEA Sites: Results From Three Waves of Consortium‐Wide Site Assessment Surveys, 2017, 2020 and 2023
Source: J Int AIDS Soc. 2026 Sep 3;29(9):e70197. doi: 10.1002/jia2.70197 (PMC13542704; doi:10.1002/jia2.70197)
Supplement: Supplementary file 1 [file JIA2-29-e70197-s001.docx]

**Supplementary Table 1. Clinic resources for adherence support and monitoring by survey timepoint and country income level**

| **Clinic resources** | **2017** | | | **2020** | | | **2023** | | | **Time trends ^b^ (2017-2023)** | |
| --- | --- | --- | --- | --- | --- | --- | --- | --- | --- | --- | --- |
|  | **LMICs** | **HICs** |  | **LMICs** | **HICs** |  | **LMICs** | **HICs** |  |  |  |
|  | **n=141** | **n=65** | **p-value^a^** | **n=148** | **n=52** | **p-value^a^** | **n=159** | **n=55** | **p-value^a^** | **LMICs** | **HICs** |
| Staffing available every day | **n (%)** | **n (%)** |  | **n (%)** | **n (%)** |  | **n (%)** | **n (%)** |  |  |  |
| Adherence counselor | 107 (75.9) | 36 (55.4) | 0.003 | 126 (85.1) | 27 (51.9) | <.001 | 133 (83.6) | 30 (54.5) | <.001 | 0.091 | 0.913 |
| Peer educators/counselors | 85 (60.3) | 12 (18.5) | <.001 | 106 (71.6) | 14 (26.9) | <.001 | 117 (73.6) | 10 (18.2) | <.001 | 0.014 | 0.983 |
| Outreach staff | 79 (56.0) | 18 (27.7) | <.001 | 73 (49.3) | 24 (46.2) | 0.694 | 89 (56.0) | 17 (30.9) | 0.001 | 0.971 | 0.640 |
| Number of pre-ART counseling sessions routinely conducted |  |  |  |  |  |  |  |  |  |  |  |
| 0 | 2 (1.4) | 18 (27.7) | <.001 ^c^ | 6 (4.1) | 14 (26.9) | <.001^c^ | 5 (3.1) | 21 (38.2) | <.001 | <.001 ^c^ | <.001^c^ |
| 1 | 65 (46.1) | 28 (43.1) |  | 63 (42.6) | 21 (40.4) |  | 102 (64.2) | 30 (54.5) |  |  |  |
| 2 or more sessions | 74 (52.5) | 19 (29.2) |  | 42 (28.4) | 8 (15.4) |  | 51 (32.1) | 4 (7.3) |  |  |  |
| Uknown/not reported | - | - |  | 37 (25.0) | 9 (17.3) |  | - | - |  |  |  |
| ART refill frequency |  |  |  |  |  |  |  |  |  |  |  |
| Monthly | 19 (13.5) | 8 (12.3) | <.001^c^ | 18 (12.2) | 8 (15.4) | <.001^c^ | 6 (3.8) | 3 (5.5) | 0.1098 | <.001 ^c^ | 0.578 ^c^ |
| Every 3 months | 103 (73.0) | 25 (38.5) |  | 105 (70.9) | 21 (40.4) |  | 66 (41.5) | 23 (41.8) |  |  |  |
| Every 6 months | 8 (5.7) | 15 (23.1) |  | 15 (10.1) | 13 (25.0) |  | 77 (48.4) | 20 (36.4) |  |  |  |
| Variable interval | 6 (4.3) | 3 (4.6) |  | 1 (0.7) | 1 (1.9) |  | 4 (2.5) | 2 (3.6) |  |  |  |
| Other/Unknown | 5 (3.5) | 14 (21.5) |  | 9 (6.1) | 9 (17.3) |  | 6 (3.8) | 7 (12.7) |  |  |  |
| Viral load testing services |  |  |  |  |  |  |  |  |  |  |  |
| No onsite viral load testing | 105 (74.5) | 21 (32.3) | <.001 | 95 (64.2) | 4 (7.7) | <.001 | 91 (57.2) | 0 (0) | <.001 | 0.002 | <.001 |
| Onsite viral testing | 36 (25.5) | 44 (67.7) |  | 53 (35.8) | 48 (92.3) |  | 68 (42.8) | 55 (100) |  |  |  |
| Turnaround time for viral load test results |  |  |  |  |  |  |  |  |  |  |  |
| 0-7 days | 29 (20.6) | 51 (78.5) | <.001 | 47 (31.8) | 42 (80.8) | <.001 ^c^ | 54 (34.0) | 48 (87.3) | <.001 | 0.0004 | 0.341 ^c^ |
| 8-14 days | 69 (48.9) | 12 (18.5) |  | 33 (22.3) | 7 (13.5) |  | 48 (30.2) | 7 (12.7) |  |  |  |
| 15-30 days | 35 (24.8) | 1 (1.5) |  | 51 (34.5) | 3 (5.8) |  | 48 (30.2) | 0 (0) |  |  |  |
| >30 days | 7 (5.0) | 0 (0) |  | 15 (10.1) | 0 (0) |  | 7 (4.4) | 0 (0) |  |  |  |
| Not available | 1 (0.7) | 1 (1.5) |  | 2 (1.4) | 0 (0) |  | 2 (1.3) | 0 (0) |  |  |  |
| Genetic testing for drug resistance |  |  |  |  |  |  |  |  |  |  |  |
| No onsite drug resistance testing | 108 (76.6) | 3 (4.6) | <.001 | 130 (87.8) | 11 (21.2) | <.001 | 127 (79.9) | 4 (7.3) | <.001 | 0.519 | 0.541 |
| Onsite drug resistance | 33 (23.4) | 62 (95.4) |  | 18 (12.2) | 41 (78.8) |  | 32 (20.1) | 51 (92.7) |  |  |  |

^a^ Chi square test, unless otherwise noted; ^b^ Cochran-Armitage trend test unless otherwise noted; ^c^ Cochran-Mantel-Haenszel test

**Supplementary Table 2. Adherence aids, support and monitoring practices by survey timepoint and country income level**

| **Clinic resources** | **2017** | | | **2020** | | | **2023** | | | **Time trends^b^ (2017-2023)** | |
| --- | --- | --- | --- | --- | --- | --- | --- | --- | --- | --- | --- |
|  | **LMICs** | **HICs** |  | **LMICs** | **HICs** |  | **LMICs** | **HICs** |  |  |  |
|  | **n=141** | **n=65** | **p-value^a^** | **n=148** | **n=52** | **p-value^a^** | **n=159** | **n=55** | **p-value^a^** | **LMICs** | **HICs** |
| ART adherence aids routinely provided to all clients | **n (%)** | **n (%)** |  | **n (%)** | **n (%)** |  | **n (%)** | **n (%)** |  |  |  |
| None | 39 (27.7) | 10 (15.4) | 0.055 | 31 (20.9) | 8 (15.4) | 0.384 | 45 (28.3) | 33 (60.0) | <.001 | 0.855 | <.001 |
| Client education materials | 49 (34.8) | 45 (69.2) | <.001 | 70 (47.3) | 28 (53.8) | 0.416 | 76 (47.8) | 18 (32.7) | 0.052 | 0.026 | <.001 |
| Pill box/blister packs | 35 (24.8) | 50 (76.9) | <.001 | 62 (41.9) | 42 (80.8) | <.001 | 40 (25.2) | 11 (20.0) | 0.439 | 0.950 | <.001 |
| Visual aids/reminders | 52 (36.9) | 15 (23.1) | 0.049 | 51 (34.5) | 19 (36.5) | 0.787 | 67 (42.1) | 5 (9.1) | <.001 | 0.331 | 0.091 |
| E-devices/reminders | 37 (26.2) | 7 (10.8) | 0.012 | 61 (41.2) | 11 (21.2) | 0.010 | 38 (23.9) | 7 (12.7) | 0.080 | 0.575 | 0.706 |
| Routine adherence support interventions for all clients | | |  |  |  |  |  |  |  |  |  |
| None | 3 (2.1) | 7 (10.8) | 0.007 | 0 (0) | 3 (5.8) | 0.003 | 23 (14.5) | 21 (38.2) | 0.000 | <.001 | <.001 |
| Individual counseling | 129 (91.5) | 55 (84.6) | 0.138 | 144 (97.3) | 46 (88.5) | 0.021 | 100 (62.9) | 30 (54.5) | 0.275 | <.001 | <.001 |
| Group counseling | 107 (75.9) | 1 (1.5) | <.001 | 103 (69.6) | 1 (1.9) | <.001 | 73 (45.9) | 2 (3.6) | <.001 | <.001 | 0.454 |
| Peer counseling/support group or ART club | 81 (57.4) | 27 (41.5) | 0.033 | 86 (58.1) | 24 (46.2) | 0.137 | 81 (50.9) | 7 (12.7) | <.001 | 0.247 | 0.001 |
| Cognitive behavioral/mental health intervention | 35 (24.8) | 34 (52.3) | <.001 | 67 (45.3) | 29 (55.8) | 0.192 | 27 (17.0) | 12 (21.8) | 0.423 | 0.092 | 0.001 |
| Other support* | 51 (36.2) | 23 (35.4) | 0.913 | 31 (20.9) | 3 (5.8) | 0.012 | 16 (10.1) | 5 (9.1) | 0.835 | <.001 | <.001 |
| Routine adherence monitoring practices | | |  |  |  |  |  |  |  |  |  |
| None | 1 (0.7) | 0 (0) | 1.000 | 0 (0) | 0 (0) | - | 0 (0) | 0 (0) | - | 0.203 | - |
| Client self-report | 109 (77.3) | 51 (78.5) | 0.853 | 122 (82.4) | 42 (80.8) | 0.788 | 128 (80.5) | 47 (85.5) | 0.412 | 0.507 | 0.330 |
| Review pharmacy pickups | 70 (49.6) | 38 (58.5) | 0.239 | 99 (66.9) | 33 (63.5) | 0.653 | 126 (79.2) | 29 (52.7) | <.001 | <.001 | 0.555 |
| Pill counts | 86 (61.0) | 10 (15.4) | <.001 | 100 (67.6) | 4 (7.7) | <.001 | 116 (73.0) | 5 (9.1) | <.001 | 0.028 | 0.256 |
| Electronic monitoring (MEMS caps) | 2 (1.4) | 1 (1.5) | 1.000 | 35 (23.6) | 3 (5.8) | 0.005 | 12 (7.5) | 0 (0) | 0.039 | 0.137 | 0.641 |
| Routine viral load monitoring | 116 (82.3) | 55 (84.6) | 0.677 | 123 (83.1) | 46 (88.5) | 0.359 | 141 (88.7) | 40 (72.7) | 0.005 | 0.117 | 0.105 |
| Adherence self-report measures |  |  |  |  |  |  |  |  |  |  |  |
| None | 32 (22.7) | 14 (21.5) | 0.006 | 26 (17.6) | 11 (21.2) | 0.001 | 15 (9.4) | 5 (9.1) | <.001 | <.001^c^ | 0.008^c^ |
| Unstructured only | 29 (20.6) | 27 (41.5) |  | 29 (19.6) | 23 (44.2) |  | 12 (7.5) | 24 (43.6) |  |  |  |
| Structured only | 62 (44.0) | 15 (23.1) |  | 75 (50.7) | 11 (21.2) |  | 57 (35.8) | 5 (9.1) |  |  |  |
| Structured and unstructured | 18 (12.8) | 9 (13.8) |  | 18 (12.2) | 7 (13.5) |  | 75 (47.2) | 21 (38.2) |  |  |  |
| Clients eligible for intensified adherence support | | |  |  |  |  |  |  |  |  |  |
| None | - | - |  | - | - |  | 0 (0) | 1 (1.8) | 0.088 |  |  |
| Self-reported adherence problems | - | - |  | - | - |  | 128 (80.5) | 53 (96.4) | 0.005 |  |  |
| Suspected adherence problems/irregular visits | - | - |  | - | - |  | 141 (88.7) | 52 (94.5) | 0.208 |  |  |
| Detectable viral loads | - | - |  | - | - |  | 108 (67.9) | 37 (67.3) | 0.929 |  |  |
| Viral load >1000 copies/mL | - | - |  | - | - |  | 48 (30.2) | 13 (23.6) | 0.354 |  |  |
| New opportunistic infections | - | - |  | - | - |  | 115 (72.3) | 27 (49.1) | 0.002 |  |  |
| New ART initiators with advanced HIV disease | - | - |  | - | - |  | 107 (67.3) | 34 (61.8) | 0.460 |  |  |

*Other support: Cash incentives, food supplements, or directly-observed treatment.

Abbreviations: ART: antiretroviral therapy; MEMS: Medication event monitoring system.

^a^Chi square test, unless otherwise noted; ^b^Cochran-Armitage trend test unless otherwise noted; ^c^Cochran-Mantel-Haenszel test.
