# Supplementary material for: Evolution of ART Medication Adherence Support and Monitoring Across IeDEA Sites: Results From Three Waves of Consortium‐Wide Site Assessment Surveys, 2017, 2020 and 2023
Source: J Int AIDS Soc. 2026 Sep 3;29(9):e70197. doi: 10.1002/jia2.70197 (PMC13542704; doi:10.1002/jia2.70197)
Supplement: Supplementary file 2 [file JIA2-29-e70197-s002.docx]

**ACKNOWLEDGEMENTS**

The following individuals led the development, design and implementation of IeDEA’s 2023 site assessment survey: Chad Achenbach, Ellen Brazier, Yanink Caro, Jessica Castilho Stephany Duda, Leslie A. Enane, Lukas Fenner, Esther Freeman, April D. Kimmel, Mark Kuniholm, Kathryn Lancaster, Fernanda Maruri, Denis Nash, Angela Parcesepe, and C. William Wester. Site investigators, clinicians and data managers who distributed and completed the survey are listed by IeDEA region, below. The authors are grateful to all these individuals for their substantive contributions, which made this analysis possible.

**ASIA-PACIFIC**

**Australia:** K Petoumenos, M Law, A Kariminia, A Han, N Roth, L Burton, J Shapiro, K Watson, J Duffy,D Russell, T Flitcroft, DJ Templeton, LM Garton, D Sowden, R Teague, E Chow, J Ong, M Gunathilake, S Hall, K Thompson, J Hoy, M Bryant, S Price, J McMahon, N Ryder, G Sweeney, E Leprince, A Carr, A Hawkes, M O'Reilly, A D Smith, A Martin Redmond, S Benn, I Woolley, J O’Bryan, K Cisera, T Korman, C Thng, R Bopage. **Cambodia:** B Ngauv, V Khol, V Bun, C Pov. **China (Hong Kong SAR):** S Chan, MP Lee, TS Kwong. **India:** R Borse, H Prasad, V Mave, S Nimkar, N Kumarasamy, S Poongulali, S Pujari, K Joshi, S Gaikwad, A Chitalikar. **Indonesia:** IKA Somia, MS Utama, AAAY Gayatri, NMDD Sukmawati, DK Wati, IB Ramajaya, D Vedaswari, A Alam, R Wisaksana, P Achdiat, S Iskandar, E Yunihastuti, S Sundari, A Widhani, B Wicaksana, N Kurniati, D Muktiarti. **Japan**: J Tanuma, H Gatanaga, H Uemura, Y Koizumi. **Malaysia:** YM Gani, TK Heng, NA Misnan, SK Chidambaram, I Azwa, S Basri, RX Ng. **Philippines:** R Ditangco, MK Pasayan, ML Mationg, N Delgado. **Singapore:** D Loy, OT Ng, Z Ferdous. **South Korea:** JY Choi, JH Kim. **Taiwan:** HP Chen, PC Wu. **Thailand:** A Avihingsanon, HMS Lwin, C Wongvoranet, C Ruengpanyathip, S Kiertiburanakul, A Phuphuakrat, L Chumla, N Sanmeema, R Chaiwarith, J Praparattanapan, K Nuket, S Khuwuwan, P Kambua, S Pongprapass, J Limlertchareonwanit. **Vietnam:** TN Pham, DTH Nguyen, DT Nguyen, TT Nguyen, NCT Nguyen, CD Do, LT Nguyen, TT Doan, LV Nguyen, PH Nguyen, TTT Giang, TTT Huong, QT Du, TT Nguyen, TTT Trang, LAT Nguyen, QN Nguyen, VTT Duong, NDH Yen. **Regional coordination and data management centers:** AH Sohn, JL Ross, B Petersen, T Suwanlerk, MG Law, K Petoumenos, A Jiamsakul, D Rupasinghe, A Kariminia.

**CARIBBEAN, CENTRAL, AND SOUTH AMERICA (CCASAnet)**

**Argentina:** Carina Cesar, Florencia Cahn, Valeria Fink, Nicolas Doudtchitzky. **Brazil:**

Sandra Wagner Cardoso, Emilia Jalil, Brenda Hoagland, Carolina Coutinho, Flavia Gomes Faleiro Ferreira, Jorge Andrade Pinto, Daisy Maria Machado, Ana Isabel de Melo Pereira Monteiro, Paulo Abrão. **Chile:** Claudia M. Cortes. **Haiti:** Vanessa Rouzier. **Honduras:** Marco Tulio Luque, Magda Chavez, Diana Varela, Isaura Herrera. **Mexico:** Brenda Crabtree-Ramirez, Jessica Mejía Castrejon, Alvaro Lopez Iniguez, Rodrigo Ville Benavides. **Peru:** Fernando Mejía, Eduardo Gotuzzo. **Regional coordination and data management center:** Jessica L. Castilho, Stephany Duda, Fernanda Maruri, C. William Wester.

**CENTRAL AFRICA IeDEA**

**Burundi:** Pelagie Nimbona, Gakima Devote, Eliane Hatungimana, Sylvain Pagnol Niragira,Thierry Nahimana, Olive Niyonkuru, Gloria Ingabire, Appolonie Nizigiyimana, Calinie Keza, Emerentienne Gahimbare, Ella-Ange Kazigamwa, Nadine Nisana, Cynthia Hitimana, Jeanine Niyonzima, Christella Twizere, Annabelle Niyongabo, Jean Marie Ntibigarura, Zacharie Ndizeye. **Cameroon:** Esther N. Neba, Denis Nsame, Ajeh Bazil, Vera Veyieeneneng, Djenabou Amadou, Eric Walter Pefura, Nicoline Ndi, Eric Ngassam, Philis Fon, Annerreke Nyenti, Judith Nasah Lainsi, Adiah Mary Akama, Anastase Dzudie, Peter Vanes Ebasone, Gabriel Mabou.

**Democratic Republic of the Congo:** Simon Kombela, Nzungani Jean-Paul, Patricia Vangu Matondo Lelo, Faustin Kitetele, Catherine Akele, Georgine Luzimbu, Eric Kiambi Makamba, Mpongo Makinda Thethe, Matthieu Musiku. **Republic of Congo:** Dominique Mahambou Nsonde, Arcel Christ Massamba Ndala, Rocide Moukouba, Merlin Diafouka, Adolphe Mafoua, Nadège Teke Bagamboula, Ursula Ingride Koukha, Nadine Mahinga. **Rwanda:** Jules Onesphore Igirimbabazi, Esperance Mujawimana, Fidele Ntarambirwa, Nicole Ayinkamiye, Ernestine Igizeneza, Providence Uwineza, Yvonne Tuyishimire, Elizaphan Nzabahimana, Emmanuel Ndamijimana, Janviere Mukankubana, Jean Baptiste Habumuremyi, Françoise Mugwaneza, Marie Alice Liliane Cyuzuzo, Marie Louise Nyiraneza, Jean Claude Habanabashaka, Sr Marie Louise Nyiransabimana, Josephine Ayinkamiye, Dieudonne Niyoyita, Liliane Tuyisenge, Rose Kwitonda, Jeanne d'Arc Mukakarangwa, Sr Epiphanie Mukashyaka, Fabien Gahunzire, Theophilla Niyigobaka, Christian Shyaka, Marie Victoire Uwambaje, Veneranda Mukarwego, Louis Gataha, Bonheur Uwakijijwe, Olive Uwamahoro, Jean d’ Amour Niyonzima, Marie Grace Ingabire, Patrick Maniragaba, Deo Gratias Mugisha, Berthilde Uwumaliya, Viateur Haburemyi, Beatrice Uwimana, Jules Ndumuhire, Sylverie Sanaso, Ruth Nyiramahirwe, Vincent Habimana, Gerald Bunani, Gilbert Maniriho, Innocent Sangwa, Fred Muyango, Wilbrold Habiyaremye, Angelique Murerwanimana, Marie Claire Uwamahoro, Olive Uwamahoro, Esperance Tuyishime, Olive Mukahirwa, Albertine Bonheur, Eugenie Mukashyaka, Florence Umugiraneza, Rosine Feza, Marie Denise Uwamahoro, Chantal Benekigeri, Jacqueline Musaninyange, Chantal Dusabe, Rose Kagaju, Shadrack Niyibizi, Marie Rosine Umugwaneza, Alex Buteera, Eric Seruyange, Thierry Habiryayo, Gad Murenzi, Gallican Kubwimana, Ben Muhoza, Athanase Munyaneza. **Regional coordination and data management center:** Ellen Brazier, Denis Nash.

**EAST AFRICA IeDEA**

**Kenya:** Mildred Kweyu, Linda Alosa, Doris Tutu Pepela, Victoria Akinyi Amonde, Moses Obuya, Kennedy Anyira Wawire, Beatrice Mayo, Josephine Mukoya, Saumu Namiso, Chirchir Benard, Levin Zeph Juma, Hillary Kiptoo, Meshack Murto, Lydia Arusei, Ann Namae, Hosea Maritim, Wilson Kirui, Fentra Sakong, Maureen Mutiembu, Moses Delewa, Sarah Cheptoo, Titus C. Rutto, Nicholas Kosgei, Felix Ambula, Josephine Chebet, Philemon Matutu, Monica Wanza, Rose Sibweche, Judith Jepyegon Kimelil, George Wafula Oduori, Kenneth Malika, Renson Ekemesi, Sadam Odhiambo, Nobrah M. Mutuku, Lydia Oloo, Koech Canicious, Wycliffe Omondi Abwao, Sarah Obatsa, Felix Ochieng, John Moracha Ogoti, Dolphine Nyaboke, Nancy Nafula, Pamela Emukule, Lameck Diero, Edwin Sang, Cosmas Apaka, Irene Wafula, John Chirchir, Violet Kisaka, Murutu J, Margaret Birgen, Janet Borosio, Abraham Kiptum Kosgei, Paul Ouma, Seth Odero, Sarah Obatsa, William Mayamba, Gabriel Netwon Alubala, Lithy David, Bonface Omalla, Cosolata Akoth, Bonface Kibet, Nelly Owiti, Lilian Ndakalu, Derick Simiyu Njofu, Elisha Chelal, Sunyai Nancy, Ariya Patrick, Ruth Rotich, Maureen Ayieko, Joyce Jelagat, Kipruto Ednah, Valentine Chebor, Victor Wezonga, Harrison Kibet, Collins Kipsang. **Tanzania:** Denna Michael, Charles Nyaga, Elcard Nunda, Regina Alphonce, Rita Lyamuya, Paul Kazyoba, Lilian Mkombachepa, Godlovenes Rubega, Jerome Lwali, Happiness Rutakulemberwa, Ruth Kamugwawa, Nicolaus Nyendikuu. **Uganda:** Nnakakawa Cissy, Ritah Namagogwe, Ahmed Ddungu, Barbara Castelnuovo, Marion Achieng, John Michael Matovu, Nanziri Allen, Nassanga Sarah J, Kyosimiire Norah, Daniel Mujunu, Nabwire Scovia, Luqman Sulaiman, Haruna, Innocent Kakeeto, Emmanuel Ssekyeru, Joseph Muleebwa, Irene Nayiga, Joan Namayanja, Brendah Nalutaaya, Sara Namembe, Esther Nakamanya, Patricia Nakato, Herbert Ssenyonga, Ruth Nazziwa Coleta, Proscovia Nantale, Joan Nayiga, Ssewanyana Tom, Namukisa Cossy, Kigozi Boniface, Rosemary Nassolo, Jane Tukashaba, Ritah Nalubega, Sandra Nabagirinya, Sylvia Nalwanga, Jumulo Nicholas , Zziwa Abiyasali, Agaba Ajaara, Julius Patrick Senoga, Kisirisa Claire, Kayovu Robert, Kamukama Brenda, Namirembe Rosemary, NIcholas Ssewankambwe, Ndagano Mercy, Semerita Muhindo, Bugembe John Nyanzi, Charles Kasozi, Phoebe Mutenyo, Matthew Ssemakadde, Winnie Muyindike , Denis Nansera, Bronia Mwiine, Patience Kusemerewa, Nakabiito Safina, Kibalama Donozio, Nalwoga Prossy, Naksaenge Cissy, Nakuya Juliet, Fred Nalugoda, Anthony Ndyanabo, Nakawooya Hadijja, Magezi Ibrahim, Orit Robert. **Regional coordination and data management center:** Kara Wools-Kaloustian, Constantin Yiannoutsos, Aggrey Semeere, Bev Musick.

**IeDEA NORTH AMERICAN AIDS COHORT COLLABORATION ON RESEARCH AND DESIGN (NA-ACCORD)**

**Canada:** John Gill, Marina Klein, Costas Pexos. **United States of America:** Michael Horberg, Cynthia S Firnhaber, Jonathan Colasanti, Julia Fleming, Ami Multani, Amanda Eldredge, Cynthia Mayer, Julie Pasternak, Mitchell Luu, Frank Palella, Ank Nijhawan, Shelly J Ramos Torres, Lymary Velazquez, Kathia Ocasio, Celestino Flores Lourdes Santiago, Angel Mayor, Jane Esteves, Rita Kelly, Jack Fuhrer, Ellen Tedaldi, Laura Bamford, Andrea Wendrow, Richard Novak, Claire Farel, Mari Kitahata, Anna Person, Theo W. Hodge Jr, Matthew B. Goetz, Greer A. Burkholder, Graham Smith, Mona Loutfy. **Regional coordination and data management center:** Keri Althoff, Richard Moore, Aimee Freeman.

**IeDEA SOUTHERN AFRICA**

**Lesotho:** Monkoe Bereng, Richard Kenyata, Tlohang Qhobela, Matlotliso Maqhai, Masefako Liotlo, Lerato Lebakeng. **Malawi:** Jacqueline Huwa, Mwagomba Crust, Damba Douglas, Ganizani Malata, Jacqueline Huwa, Eric Mtemang'ombe. **Mozambique:** Idiovino Rafael. **South Africa:** Zakhona Ndlovu, Nosisa Sipambo, Geoffrey Fatti, Nontando Xaba, Bongi Cele, Sibongokuhle Ntombela, Jonathan Euvrard, Brian Eley, Hans Prozesky, Thalia Ferreira, Nicola Van Dongen, Cuma Koncwana, Chantel Schreuder. **Zambia:** Gerald Mungwa, Gift Chongo, Precious Kakvugu, Harriet Chabala, Fiennel Moonde, Leah Shapi, Dora Mwaba, Josephine C Sithole, Derrick Mwenda Filumba. **Zimbabwe:** Kumbirai Pise Quarter, Cleophas Chimbetete. **Regional coordination and data management center:** Morna Cornell, Per von Groote, Mary-Ann Davies, Matthias Egger.

**IeDEA WEST AFRICA**

**Benin:** Gael Ayihounton, Tounkara Lidie, Bokossa Laurelle, Zannou Marcel Djimon, Léhila Bagnan Tossa. **Burkina Faso:** Armel Poda, Gbolo Pooda, Caroline Yonaba. **Côte d’Ivoire:**

Eugène Messou, N'Gbeche Marie Sylvie, Albert Kla Minga, Stéphane Kouadio N'Goran, Henri Chenal, Kouakou Kouadio, Daple Beugre N'Guessan Jacques, Amorissani Folquet, Eboua Tanoh. **Ghana:** Joycelyn Assimeng Dame, Islynn Gloria Aggrey, Vincent Ganu. **Mali:** Yacouba Aba Coulibaly. **Nigeria:** Oliver Ezechi, Agatha N. David, Abdulrasheed A. Oba, Abdulrasheed.  **Togo:** Elom Takassi, Mensah Ephrem. **Regional coordination and data management center:** Thierry Tiendrebeogo, Karen Malateste, Antoine Jaquet, Valeriane Leroy.
